# Pressurized Liquid Extraction of Antioxidant and α-Amylase-Inhibitory Compounds from Red Seaweed Using Water–Ethanol Mixtures

**DOI:** 10.3390/molecules29215018

**Published:** 2024-10-24

**Authors:** Nils Leander Huamán-Castilla, Erik Edwin Allcca-Alca, Frank Hervas Nina, Nilton Cesar León-Calvo, Franz Zirena Vilca, Yesica Luz Vilcanqui Chura

**Affiliations:** 1 Escuela Profesional de Ingeniería Agroindustrial, Universidad Nacional de Moquegua, Prolongación Calle Ancash s/n, Moquegua 18001, Peru; eallccaa@unam.edu.pe (E.E.A.-A.); frankhervas17@gmail.com (F.H.N.); nleonc@unam.edu.pe (N.C.L.-C.); yvilcanquic@unam.edu.pe (Y.L.V.C.); 2 Laboratorio de Tecnologías Sustentables para la Extracción de Compuestos de Alto Valor, Instituto de Investigación para el Desarrollo del Perú (IINDEP), Universidad Nacional de Moquegua, Moquegua 18001, Peru; 3 Laboratorio de Contaminantes Orgánicos y Ambiente, Instituto de Investigación para el Desarrollo del Perú (IINDEP), Universidad Nacional de Moquegua, Moquegua 18001, Peru; fzirenav@unam.edu.pe

**Keywords:** red seaweed, antioxidant compounds, solvent composition, pressurized liquid extraction, α-amylase inhibition

## Abstract

Red seaweeds from the coastal shores of Ilo (Peru) are a natural source of high-value compounds beneficial to health due to their high antioxidant capacity. Thus, this work evaluated the effect of water–ethanol mixtures (0, 15, and 30%; *v*/*v*) at high temperatures (90, 120, and 150 °C) on the polyphenol content, antioxidant capacity, and polyphenols profile of red seaweed (*Chondracanthus chamissoi*) during a pressurized liquid extraction process, whose parameters were set at 10 atm, with a single cycle of extraction and a volume of 150%. An increase in temperature and ethanol had a positive effect on antioxidant compounds. Thus, the best processing conditions were established at 150 °C and 30% ethanol, allowing for the extraction of a high polyphenol content (2.04 mg GAE/g dw) and antioxidant capacity (IC50: 7.46 mg/mL, ORAC: 148.98 μmol TE/g dw). High ethanol concentrations (30%) effectively recovered phenolic acids, flavonols, and phlorotannins for the polyphenols profile. However, the use of pure water was more effective in recovering flavonols. Interestingly, using pure water as an extraction solvent at high temperatures allowed for a more significant inhibition of the α-amylase enzyme than water–ethanol mixtures under the same conditions. Finally, the results can be utilized for future industrial scaling and the potential utilization of extracts in developing diabetes treatments.

## 1. Introduction

Between 2015 and 2020, Peru experienced a remarkable surge in its seaweed exports, the growth of which increased by approximately 216% [1,2]. In particular, this period saw a particularly noteworthy expansion in the export of red seaweed (*Chondracanthus chamissoi*), which increased by about 140%, finding its primary markets in countries like China, Canada, and the United States, whose sales reached a value of approximately USD 5 million [1,2]. This international demand for red seaweed can be attributed to its rich composition of carbohydrates (<60%), proteins (17–44%), and lipids (<45%), as well as its high carrageenan, amino acids, and unsaturated fatty acids concentrations [3]. These compounds are used to formulate foods with a high nutritional value and a long shelf life [4]. However, there is a significant price discrepancy between the dried seaweed sold to the international market (USD 200 per tn) and the price paid to local artisanal fishermen or small associations for fresh seaweed (USD 25 per tn) [5]. Thus, it is necessary to carry out new studies to identify and extract specific antioxidant compounds from seaweed to enhance its value and improve the economic well-being of families dependent on this industry.

Red seaweeds are a natural source of antioxidant compounds known as polyphenols. These compounds can be classified as phenolic acids, flavanols, flavonols, bromophenols, and phlorotannins, which present bioactive and nutraceutical properties, making them attractive to the pharmaceutical industry for their potential to treat oxidative stress-related diseases [6,7]. For example, gallic acid, a phenolic acid, presents antioxidant, anti-inflammatory, and antineoplastic properties [8]. Flavanols are catechins that can inhibit glutathione peroxidases (GPO), reducing oxidative damage to the colon [9]. Meanwhile, phlorotannins, like phloroglucinol, are used as a defense mechanism as they neutralize oxidizing agents in our bloodstream [10]. In addition, the economic value of these polyphenols, depending on their purity and the specific type of polyphenol, may vary between USD 1200 and 33,000 per gram [11]. These considerations represent an opportunity to adopt sustainable extraction technologies to recover polyphenols from red seaweeds.

Traditional extraction methods used to recover polyphenols under atmospheric conditions have been shown to be inefficient due to their consumption of excessive amounts of time and energy, as well as the use of unfriendly solvents (acetone, methanol, hexane), which render the extracts unsuitable for food-grade applications [4,12,13]. Although conventional extraction methods using water–ethanol mixtures have been developed to recover polyphenols from seaweed, the long processing times (>2 h) and low yields limit their scaling up to an industrial level. [14,15]. In contrast, alternative extraction methods like pressurized liquid extraction (PLE), ultrasound-assisted extraction (UAE), and microwave extraction have demonstrated efficiency in recovering polyphenols, yielding results that are 2 to 3 times greater than those achieved with traditional methods [13,16,17]. In this sense, Santos et al. [18,19] found that the shorter processing times and higher temperatures in ELP lead to lower operational costs compared to alternative technologies such as ultrasound-assisted extraction (UAE) and the Soxhlet method.

Pressurized Liquid Extraction (PLE) is an alternative technology that operates at high pressures (~10 atm) and elevated temperatures (90–250 °C); these conditions allow the solvent to maintain a liquid state, enhancing its solvation capability as a result of decreased polarity [20,21]. In addition, when combined with food-grade solvents (ethanol and glycerol), this technology presents a higher efficiency in recovering antioxidant compounds than pure water under the same conditions [17,22]. However, the effect of PLE on extracting polyphenols from seaweeds remains underexplored, necessitating further research to optimize the processing parameters.

Type 2 diabetes mellitus is characterized by elevated blood glucose levels resulting from inadequate insulin production [23]. In Peru, ~1.3 million people between 20 and 79 years old are afflicted with this disease [24]. Thus, local authorities have prioritized the distribution of synthetic medications like acarbose to treat type 2 diabetes. Interestingly, some studies have demonstrated that polyphenols can inhibit specific enzymes like α-amylases and α-glucosidases through their hydroxyl groups and catalytic site interactions [25]. However, the inhibiting capacity of the extracts was relatively low [26,27]. Under these considerations, this study aims to evaluate mixtures of water–ethanol combined with high temperatures (90–150 °C) at 10 atm to establish an efficient recovery of specific polyphenols from red seaweed as a new alternative for treating type 2 diabetes. This would enhance its economic value and contribute to the sustainability of seaweed exploitation.

## 2. Results and Discussion

### 2.1. Total Polyphenol Content (TPC)

**Figure 1:**
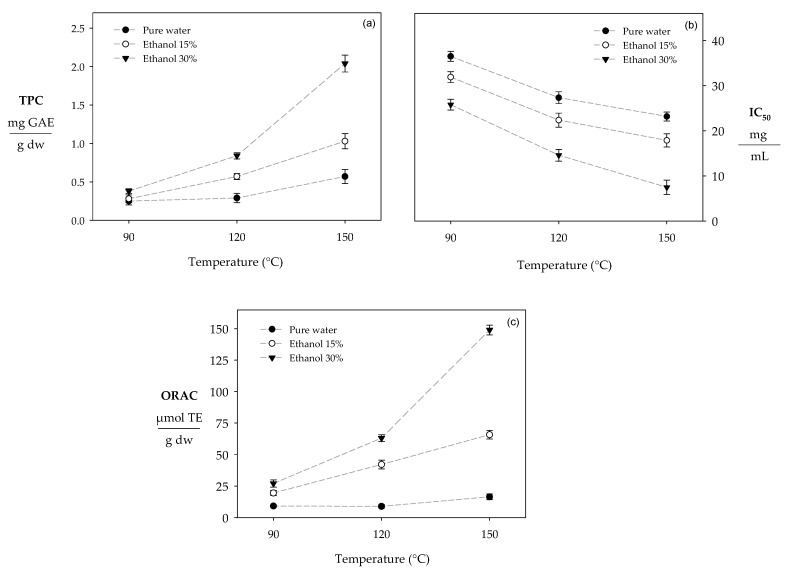
Effect of temperature and solvent composition on antioxidant compounds. Note: (**a**) TPC; (**b**) IC_50_; (**c**) ORAC.

According to our results, the higher the temperature and ethanol composition, the more the total polyphenol content increases, with values ranging between 0.25 and 2.04 mg GAE/g dw (Figure 1a). Regardless of the solvent composition, temperature increases during the PLE process improved the recovery of polyphenols. For example, when the temperature increased from 90 to 150 °C, the TPC was enhanced by 2, 3.5, and 5.4 times with pure water, 15% ethanol, and 30% ethanol, respectively (Figure 1a).

### 2.2. Antioxidant Activity

For the DPPH method, using ethanol at high temperatures during the PLE process allowed us to obtain extracts with significant antioxidant capacity values (IC_50_: 14.57–36.49 mg/mL) (Figure 1b). However, it is important to mention that the lower the IC_50_ value, the greater the extract’s antioxidant capacity (IC50). Thus, several studies mention that the IC_50_ value is inversely proportional to the polyphenol content. According to our results, when the temperature increased from 90 to 150 °C, the IC_50_ was improved by 36%, 43%, and 71% with pure water, 15% ethanol, and 30% ethanol, respectively (Figure 1b).

For the ORAC method, this analysis assesses the ability of polyphenols to reduce biological radicals known as peroxyls, one of the body’s most common reactive oxygen species [28]. Thus, when extracts have a high content of polyphenols, the antioxidant capacity of the extract is greater [29]. The antioxidant capacity evaluated by ORAC in the extracts obtained increased with temperature and ethanol concentrations. For example, an increase from 90 to 150 °C improved the antioxidant capacity by 2.8, 3.4, and 5.5 times with pure water, 15% ethanol, and 30% ethanol, respectively (Figure 1c).

### 2.3. Effect of Water-Ethanol Mixtures at High Temperatures on the Polyphenol Profile

#### 2.3.1. Phenolic Acids

**Figure 2:**
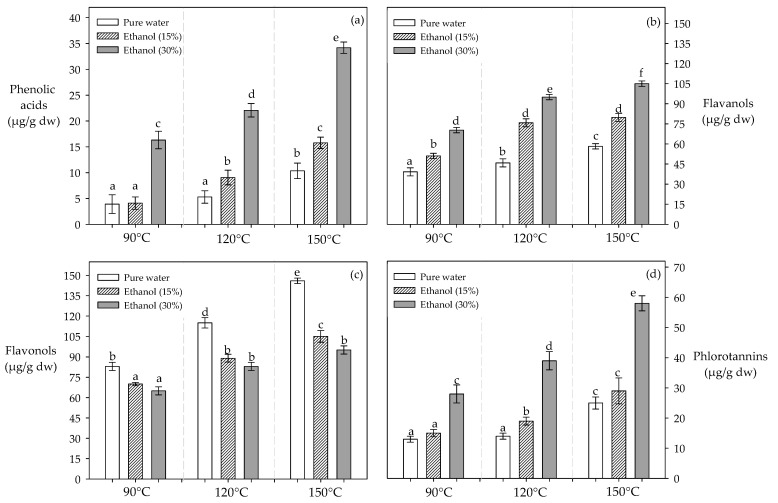
Effect of solvent composition and temperature on the recovery of polyphenol families. Note: (**a**) Phenolic acids; (**b**) Flavanols; (**c**) Flavonols; (**d**) Phlorotannins. Different letters indicate statistically significant differences (*p* < 0.05) for each extraction process.

The phenolic acid performance improved with the increase in ethanol and temperature during the PLE process (Figure 2a). For example, when the temperature increased from 90 to 150 °C, the extraction capacity of phenolic acids improved by approximately 2, 2.6, and 3.8 times with 0%, 15%, and 50% ethanol, respectively (Figure 2a). According to our results, the best processing conditions were established at 150 °C with 30% ethanol (Figure 2a). This allowed for the recovery of specific phenolic acids such as gallic, vanillic, and caffeic, and the predominant phenolic acid was gallic acid with 15.26 μg/g dw (Table 1).

#### 2.3.2. Flavanols

Under subcritical conditions (P: ~10 MPa), high temperatures and ethanol concentration improved the recovery of these compounds. Thus, the highest recovery of flavanols was achieved at 150 °C, with 30% ethanol (105.02 μg/g dw) (Figure 2b). In our study, the best process conditions (150 °C–30% ethanol) allowed us to recover significant concentrations of flavanols such as catechin, epicatechin, and procyanidins (B2 and A2) with 13.46, 24.53, 25.88, and 30.15 μg/g dw, respectively (Table 1).

#### 2.3.3. Flavonols

Although high extraction temperatures improved the extraction of flavonols, an increase in the ethanol concentration decreased the recovery of these compounds (Figure 2c). For example, a change from 0 to 30% ethanol at 150 °C reduces the recovery of flavonols by 69% (Figure 2c). The highest recovery of flavonols was achieved at 150 °C with pure water, recovering high concentrations of quercetin, kaempferol, and rutin (Table 1).

#### 2.3.4. Phlorotannins

Like phenolic acids and flavanols, the recovery of phlorotannins increased with the amount of ethanol and the temperature, and their concentrations ranged from 12.09 to 58.67 µg/g dw (Figure 2d).

### 2.4. Effect of Water-Ethanol Mixtures at High Temperatures on α-Amylase

**Figure 3:**
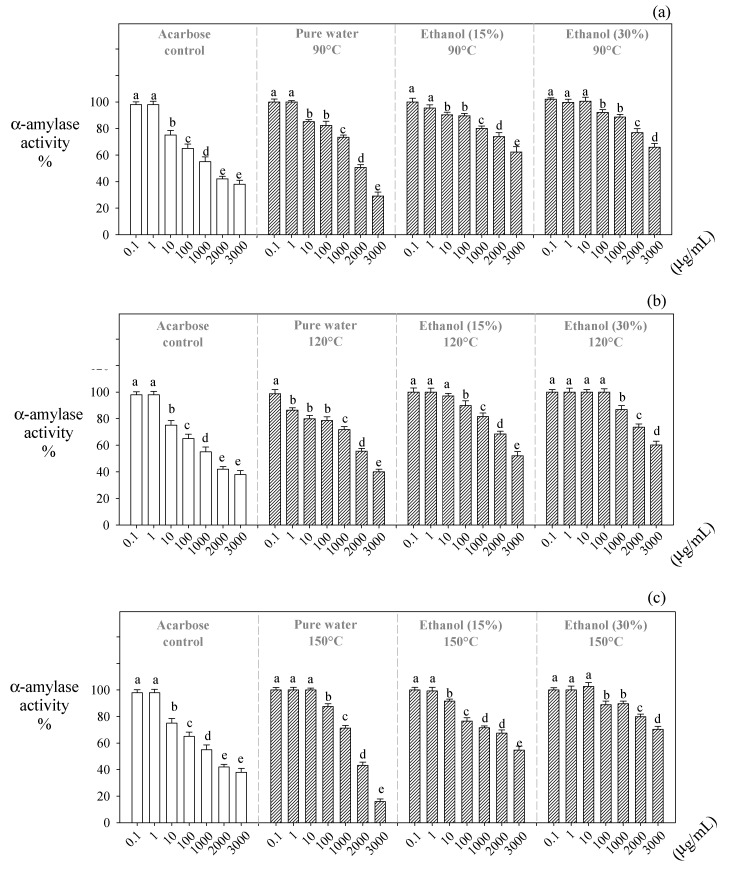
Effect of the extracts obtained on α-amylase activity. Note: (**a**) 90 °C; (**b**) 120 °C; (**c**) 150 °C. Different letters indicate statistically significant differences (*p* < 0.05) for each extraction process.

According to our results, pure water was more effective than water–ethanol mixtures at inhibiting the amylase enzyme’s activity in obtaining polyphenolic extracts from red algae. Pure water reduced amylase activity by 30%, 40%, and 16% at 90, 120, and 150 °C, respectively (Figure 3). On the other hand, Acarbose reduced the enzyme activity by up to 38%, showing the effectiveness of water extracts obtained compared to this drug (Figure 3).

## 3. Discussion

### 3.1. Total Polyphenol Content (TPC)

Although there are no reports on the positive effect of high temperatures under subcritical conditions (10 atm) on the recovery of total polyphenols from red seaweeds, other studies have recovered these compounds from other plant matrices. For example, Otero et al. [30] showed that when the temperature increases from 80 to 160 °C using water–ethanol mixtures (50%), the recovery of polyphenols from *L. ochroleuca* seaweed improved by 39%. Allcca-Alca et al. [31] reported that an increase from 90 to 150 °C combined with 15% ethanol increases the total polyphenol content by ~3 times from grape pomace, while Huaman-Castilla et al. [22] demonstrated that the use of pure water combined with an increase from 70 to 130 °C improved the extractability of polyphenols from discarded blueberries by four times. Elevated temperatures also increase the kinetic energy of the solvent molecules, which enhances mass transfer and promotes better penetration into the cellular structures of the plant material [17,22,31]. This process disrupts the cell walls and helps release bound polyphenols of high molecular weight more effectively [32]. Moreover, higher temperatures can reduce the solvent’s viscosity, allowing for a more efficient solvent flow and interaction with the target compounds [17,21]. On the other hand, high-temperature extraction (>120 °C) can lead to the degradation of certain sensitive polyphenols; it can also promote the formation of new antioxidant compounds, which can enhance the overall antioxidant capacity of the extract [33,34].

On the other hand, an increased ethanol concentration improved the recovery of polyphenols from the extracts obtained (Figure 1a). For example, an increase from 0 to 30% ethanol at 150 °C enhanced the extraction of polyphenols by 64% (Figure 1a). Tierney [35] demonstrated that an increase in ethanol concentration from 0 to 80% at 60 °C under subcritical conditions improved the recovery of polyphenols by 37% from brown seaweed (*F. Spiralis*). On the other hand, under atmospheric conditions, Fu et al. [14] reported that a change from 0 to 50% ethanol at 30 °C improves the recovery of polyphenols from red seaweeds by 60%. This positive effect of ethanol is because polyphenols are compounds with a more remarkable ability to establish interactions with solvents of intermediate polarity than ethanol [36]. Ethanol presents a hydroxyl group (polar fraction) and a methyl group (non-polar fraction), which can establish intermolecular interactions with the functional groups of polyphenols (hydroxyl group and aromatic rings). In contrast, pure water only presents hydroxyl groups in its chemical structure [37]. Moreover, ethanol helps reduce the solvent’s surface tension, improving its penetration into the plant matrix and promoting a more efficient mass transfer during extraction [38].

### 3.2. Antioxidant Activity

For DPPH (Figure 1b), Gan and Baroutian [39] reported similar behavior under subcritical conditions and found that an increase from 120 to 150 °C reduces the IC_50_ in extracts obtained from seaweed by 65% (*Undaria pinnatifida*). Mamani-Pari et al. [38] reported that an increase from 50 to 70 °C combined with pure water reduced the IC_50_ value of the red peel prickly pear by 71%. Huaman-Castilla et al. [17] demonstrated that an increase from 50 to 70 °C combined with pure water improves the antioxidant activity expressed as an IC_50_ value. In this regard, temperature increases accelerate the solvent molecules (kinetic energy), favoring the cellular matrix’s breakdown and subsequent release of polyphenols [22]. At subcritical temperatures (between 50 °C and 150 °C), the increased thermal energy can reduce the ethanol polarity, decreasing its ability to form hydrogen bonds (α) from 0.83 to 0.51 [40]. These conditions increase the nonpolar interaction between aromatic groups of polyphenol and methyl groups of ethanol. Thus, a higher concentration of polyphenols in the extract will increase the antioxidant capacity of the extracts. Consequently, the IC_50_ value will be lower.

For the ORAC analysis (Figure 1c), several studies have reported that temperature increases combined with water–ethanol mixtures during a subcritical extraction process favor the recovery of polyphenols due to the reduction in solvent polarity and the breakdown of the plant matrix, thereby improving the capacity of the extracts expressed as the ORAC value [27,31,41]. Mamani-Pari et al. [38] reported that using pure water combined with increasing the temperature from 50 to 70 °C improves the antioxidant capacity by 50%. Huaman Castilla et al. [22] found that an increase from 50 to 70 °C increased the antioxidant capacity by 3, 1.5, and 1.8 times with pure water and 15% and 30% ethanol, respectively. On the other hand, Plaza et al. [42] reported that when the temperature exceeds 120 °C under subcritical conditions, the formation of antioxidant compounds as a product of the Maillard reaction is favored. Consequently, the antioxidant capacity of the extracts is enhanced.

### 3.3. Effect of Water-Ethanol Mixtures at High Temperatures on the Polyphenol Profile

#### 3.3.1. Phenolic Acids

According to the results reported in Figure 2a, a similar behavior was reported by Huaman-Castilla et al. [20], who noted that, with an increase from 90 to 150 °C, the recovery of phenolic acids improved by ~9, ~12, and ~19 times with 15%, 32.5%, and 50% ethanol, respectively. Allcca-Alca et al. [33] found that using 20 and 60% ethanol at 100 °C increased the recovery of phenolic acids by 19%. Using ethanol as a cosolvent combined with high temperatures during the PLE process decreases the solvent’s polarity, favoring non-polar interactions between polyphenols and solvent [27].

#### 3.3.2. Flavanols

Similar to the behavior of phenolic acids, various authors have demonstrated the affinity of these compounds for water–ethanol mixtures due to polar and non-polar interactions [17,20,21]. Huaman-Castilla et al. [20] reported that high temperatures (150 °C) with 32.5% ethanol allowed for the recovery of the highest yields of flavanols (92.68 μg/g dw). Similarly, Allcca-Alca [31] found that the use of high ethanol composition (60%) at 100 °C recovered the highest concentration of flavanols (46.05 μg/g dw). Flavanols exhibit a strong affinity for ethanol during extraction processes, primarily due to the interaction between the hydroxyl (–OH) groups of polyphenol and ethanol. Moreover, ethanol’s dual polarity enables it to effectively interact with both the polar functional groups of flavonols and their non-polar aromatic rings [17,22,32].

#### 3.3.3. Flavonols

Huaman-Castilla et al. [20] reported that 15 to 50% ethanol at 150 °C disfavors the recovery of these compounds by 19% from grape pomace extracts. Mamani-Pari et al. [38] reported that an increase from 30 to 60% ethanol reduced the recovery of these compounds from red peel prickly pear by 52%. It is likely that the presence of carbonyl groups in the structure of flavonols enhances their solubility in water. Thus, as the ethanol concentration increases, ethanol–water interactions are favored over interactions with polyphenols, which would decrease the solubility of flavonols, reducing their recovery during extraction.

#### 3.3.4. Phlorotannins

Erpel et al. [27] and Pacheco et al. [26] reported in brown algae that, although there is a presence of families of phenolic acids, flavonols, and flavonols, there is also a significant concentration of phlorotannins. The presence of multiple hydroxyl (–OH) groups in phlorotannins enhances their ability to form hydrogen bonds with ethanol, improving its solubility. Moreover, the amphiphilic nature of ethanol allows it to interact favorably with non-polar regions of phlorotannins (aromatic rings) [26,27,43]. The antioxidant capacity of these polyphenols (phlorotannins) is estimated to be 2 to 3 times better than other specific families [12,27].

### 3.4. Effect of Water-Ethanol Mixtures at High Temperatures on α-Amylase

Although some studies have reported that high-molecular-weight polyphenols (procyanidins) are more effective in inhibiting the α-amylase enzyme [25,44], it is necessary to consider that these compounds are more soluble in water–ethanol mixtures compared to those extracts obtained with pure water [45]. In pure water, monomeric polyphenols like phloroglucinol, gallic acid, and quercetin are more soluble and stable, inhibiting α-amylase. At the same time, procyanidins may precipitate or form complexes that reduce their solubility, diminishing their inhibitory capacity. Phloroglucinol presents hydroxyl groups, which can form hydrogen bonds with polar amino acid residues (serine, threonine, aspartate, and glutamate) in the active site of α-amylase, inhibiting its enzymatic activity. At the same time, the aromatic rings can contribute to hydrophobic interactions with non-polar residues in the enzyme’s active site, blocking access to the substrate (starch) and inhibiting its hydrolysis [25,46,47].

## 4. Materials and Methods

### 4.1. Samples

The collection of seaweed was carried out in June 2023 and conducted according to the protocol proposed by Vilcanqui et al. [6]. In total, 10 kg of fresh red seaweed was harvested from the coastal shores of Ilo, Peru (coordinates 17°38′38″ S, 71°20′47″ W). The collected samples were placed in a cooler with cooling gels to maintain them at 10 °C and ensure their preservation during transportation to the laboratory. The samples were rinsed with distilled water to remove sand and salt. The samples were then frozen at −20 °C. After freezing, they were milled (particle diameter: ~1 mm) and stored at −20 °C before the extractions.

### 4.2. Chemicals and Reagents

Sigma Aldrich (St. Louis, MO, USA) provided products and chemicals like Folin–Ciocalteu’s reagent, sodium carbonate, DPPH, AAPH, and Trolox. PanReac AppliChem ITW Reagents (Darmstadt, Germany) provided organic solvents like ethanol, methanol, and acetone. Additionally, JT Baker Chemical Co. (Phillipsburg, NJ, USA) provided potassium phosphate buffers. Finally, Sigma Aldrich also provided standards for polyphenols such as caffeic acid, vanillic acid, catechin, and others.

### 4.3. Extraction Technique

The extraction was carried out according to the method proposed by Huaman-Castilla et al. [17], with some modifications. In brief, 10 g of the red seaweed was mixed with quartz sand and placed in a 100 mL extraction cell into a pressurized liquid extraction system (ASE 150, Dionex, Thermofisher, San Jose, CA, USA). The process conditions (solvent composition and temperature) were established using previous work carried out by Erpel et al. [27] and Pacheco et al. [26]. In this sense, water–ethanol mixtures (0–30%) at high temperatures (90–150 °C), whose conditions were set to 10 atm, one extraction cycle, 150% washing volume, 250 s nitrogen purge time, and 5 min static extraction time, were used to obtain a matrix/extractant ratio of 1:10. Then, the extracts were preserved in amber vials at −20 °C for analysis.

### 4.4. Total Polyphenol Quantification

The total polyphenol content in extracts was determined using the Folin–Ciocalteu method, as described by Singleton and Rossi [48]. A mixture containing 3.75 mL of distilled water, 0.5 mL of extract, 0.25 mL of Folin–Ciocalteu reagent (1 N), and 0.5 mL of sodium carbonate solution (10% *w*/*v*) was prepared. This mixture was incubated at room temperature in the dark for one hour. Subsequently, absorbance was measured at 765 nm using a UV-Vis Genesys 150 spectrophotometer (Thermofisher, San Jose, CA, USA). The results were expressed as gallic acid equivalents per gram of dry weight.

### 4.5. Determining Antioxidant Efficacy (DPPH)

The antioxidant capacity of the extracts was determined using the DPPH (2,2-diphenyl-1-picrylhydrazyl) radical inhibition method, which was reported by Brand-Williams et al. [49]. In summary, 0.1 mL of the extract was mixed with 3.9 mL of DPPH solution (50 µM). Then, the solution was incubated at room temperature in the dark for 30 min. The reduction in the DPPH radical was measured at 517 nm using a UV-Vis spectrophotometer (Genesys 150, Thermofisher, San Jose, CA, USA). Methanol was used as a positive control, while the methanolic DPPH solution was the negative control. The results were expressed as IC50 factor (mg/mL), the effective extract concentration required to inhibit 50% of the DPPH radical activity.

### 4.6. Antioxidant Capacity Evaluated by ORAC Assay

The ORAC analysis of the extracts was carried out using a microplate reader (Synergy/HTX, Biotek Instruments Inc., Winooski, VT, USA); this analysis was performed according to the methodology proposed by Chambia et al. [50]. In brief, 75 mM PBS buffer solution at 7.4 of pH was prepared. Fluorescein (55 nM), AAPH (153 mM), and Trolox were used as reference standards (8, 16, 24, 32, and 40 µM), and each sample was diluted with PBS buffer. Then, 25 µL of the Trolox sample and blank (PBS buffer) were added into a 96-well black microplate. The equipment automatically injected 250 µL of fluorescein into each well, and the mixture was incubated at 37 °C for 10 min. Subsequently, 25 µL of AAPH was injected into each well. Then, fluorescence was measured at 485 nm (excitation) and 520 nm (emission) every minute for 50 min. Finally, the ORAC values were calculated using the area under the curve and were expressed as µmol TE/g dry weight.

### 4.7. α-Amylase Activity

The method for measuring the inhibition of α-amylase activity was adapted from Huaman-Castilla et al. [45]. Initially, each extract was dried using nitrogen gas, redissolved in DMSO (dimethyl sulfoxide), and then filtered to prepare a 10 mg/mL sample stock solution. The experimental dilutions ranged from 0.1 to 3000 µg/mL in phosphate buffer (pH 6.9). For the assay, 100 µL of each dilution was mixed with 100 µL of 1% starch solution in a 20 mm sodium phosphate buffer (pH 6.9) and incubated at 25 °C for 10 min. Afterward, 100 µL of porcine pancreatic α-amylase solution (0.5 mg/mL) was added to each sample, and incubation continued for an additional 10 min at 25 °C. The reaction was terminated by adding 200 µL dinitro salicylic acid reagent, and the mixture was heated at 100 °C for 5 min. Afterward, 50 µL from each sample was transferred to a 96-well microplate, diluted with 200 µL of water per well, and the absorbance was measured at 540 nm to determine the enzymatic activity.

$$Amylase\;Activity=\frac{Absorbance\;of\;extract}{Absorvance\;of\;control}\times 100$$

The control is the enzyme–substrate reaction in the absence of inhibitors. The effect of the pharmacological inhibitor, acarbose, was also determined following the same protocol previously described.

### 4.8. Polyphenol Profiling

The methodology proposed by Huamán-Castilla et al. [17] was employed with some modifications to quantify specific polyphenols. Before the chromatographic analysis, sample preparation was conducted through solid-phase extraction (SPE), using C-18 cartridges of 5 mL, 500 mg (SiliCycle, Québec City, QC, Canada). Subsequently, an ultra-high-performance liquid chromatography (UHPLC) coupled with a diode-array detector (DAD) (Agilent 1290 Infinity II, Agilent Technologies, Santa Clara, CA, USA) was utilized to inject 2 µL of the prepared mixture. Compound separation was achieved using a reverse-phase analytical column (Poroshell EC-C18, 2.1 mm × 150 mm × 1.9 µm) at 30 °C. The mobile phase consisted of Milli-Q water with 0.1% formic acid (A) and acetonitrile with 0.1% formic acid (B), using a gradient elution: 0 min 95% A—5% B, 15 min 60% A—40% B, and 18 min 95% A—5% B at a flow rate of 0.3 mL/min. The analyses were conducted in triplicate, and the results were expressed in µg of specific polyphenol per gram on a dry-weight basis.

### 4.9. Statistical Analysis

The experimental design comprises a complete factorial design (3 × 3) to evaluate the effect of study factors (temperature and solvent) on the response variables (total polyphenols, antioxidant capacity, and α-amylase activity). Subsequently, an Analysis of Variance (ANOVA) was performed to determine if significant differences existed between facto and interactions (*p*-value < 0.05). Tukey’s test (*p*-value < 0.05) was performed for pairwise comparison.

## 5. Conclusions

High temperatures (150 °C) and ethanol concentrations (30%) allowed for the recovery of polyphenolic extracts with a high antioxidant capacity. The chemical structure of ethanol has a polar (hydroxyl) and non-polar fraction (methyl). Although high temperatures can effectively recover specific families of polyphenols, the solvent composition allowed us to obtain a selective process. Although the high temperatures used effectively recovered specific families of polyphenols, the solvent composition allowed for a selective recovery of particular polyphenols. High concentrations of ethanol (30%) effectively recovered phenolic acids, flavonols, and phlorotannins. Conversely, the use of pure water was more effective in recovering flavonols. Due to their high monomer content, extracts obtained with pure water were more effective at inhibiting amylase activity than those obtained with water–ethanol mixtures. These results suggest the potential for scalability for industrial applications and open the door to developing functional foods and nutraceuticals that utilize these beneficial compounds.

## Figures and Tables

**Table 1:** The polyphenol profile of the extracts obtained.

Description	Pure water			Ethanol (15%)			Ethanol (30%)		
	90 °C	120 °C	150 °C.	90 °C	120 °C	150 °C	90 °C	120 °C	150 °C
	Mean ± DS	Mean ± DS	Mean ± DS	Mean ± DS	Mean ± DS	Mean ± DS	Mean ± DS	Mean ± DS	Mean ± DS
**Phenolic acids** (µg/g dw)									
Gallic	2.83 ^a^ ± 0.34	3.08 ^b^ ± 0.12	5.63 ± 0.41 ^d^	4.11 ^c^ ± 0.33	5.16 ^d^ ± 1.02	8.86 ^e^ ± 1.52	7.23 ^e^ ± 1.88	8.66 ^e^ ± 1.23	15.26 ^f^ ± 2.32
Caffeic	ND	ND	0.47 ^a^ ± 0.07	ND	0.89 ^b^ ± 0.06	1.35 ^c^ ± 0.22	3.44 ^d^ ± 0.78	4.98 ^d^ ± 0.79	7.78 ^e^ ± 1.71
Vanillic	ND	2.22 ^a^ ± 0.09	4.35 ^c^ ± 0.61	ND	3.01 ^b^ ± 0.44	6.42 ^d^ ± 0.71	5.67 ^d^ ± 1.03	8.73 ^e^ ± 0.81	11.14 ^f^ ± 1.02
**Flavanols** (µg/g dw)									
Catechin	2.11 ^a^ ± 0.27	3.67 ^b^ ± 0.12	5.26 ^c^ ± 1.05	4.11 ^c^ ± 1.07	12.09 ^d^ ± 1.55	19.05 ^f^ ± 3.45	10.18 ^d^ ± 0.81	13.89 ^d^ ± 0.83	15.46 ^e^ ± 1.01
Epicatechin	13.45 ^a^ ± 1.08	16.78 ^b^ ± 1.45	20.21 ^c^ ± 1.12	15.23 ^b^ ± 1.98	21.87 ^c^ ± 1.98	25.03 ^d^ ± 1.88	19.23 ^c^ ± 1.04	24.53 ^d^ ± 2.78	28.53 ^e^ ± 2.77
Procyanidin B2	6.77 ^a^ ± 1.11	7.12 ^a^ ± 1.89	10.85 ^b^ ± 1.15	12.33 ^b^ ± 1.33	18.44 ^c^ ± 2.89	22.34 ^c^ ± 1.76	16.78 ^c^ ± 1.11	27.89 ^d^ ± 1.47	25.88 ^d^ ± 2.99
Procyanidin A2	16.88 ^a^ ± 1.21	18.33 ^a^ ± 1.07	22.93 ^b^ ± 1.81	19.36 ^b^ ± 1.78	23.41 ^b^ ± 1.08	27.37 ^c^ ± 1.98	23.15 ^b^ ± 1.75	30.56 ^c^ ± 2.47	34.15 ^c^ ± 3.22
**Flavonols** (µg/g dw)									
Quercetin	6.89 ^a^ ± 1.38	17.89 ^d^ ± 1.11	23.15 ^c^ ± 1.55	4.35 ^a^ ± 1.55	10.92 ^b^ ± 1.09	17.89 ^d^ ± 1.78	9.09 ^b^ ± 1.05	14.78 ^c^ ± 1.61	16.15 ^d^ ± 1.56
Kaempferol	8.96 ^b^ ± 1.82	18.66 ^c^ ± 2.29	27.60 ^d^ ± 1.21	8.18 ^b^ ± 1.22	16.02 ^c^ ± 1.44	18.13 ^c^ ± 1.66	5.92 ^a^ ± 0.96	14.03 ^c^ ± 1.18	15.60 ^c^ ± 1.09
rutin	67.87 ^a^ ± 2.44	81.34 ^b^ ± 3.13	95.70 ^c^ ± 7.92	55.54 ^a^ ± 7.41	65.66 ^a^ ± 4.28	69.01 ^a^ ± 7.27	51.67 ^a^ ± 5.34	59.77 ^a^ ± 3.33	62.70 ^a^ ± 5.77
**Phlorotannins** (µg/g dw)									
Phloroglucinol	13.09 ^a^ ± 2.56	15.67 ^a^ ± 1.78	28.45 ^c^ ± 2.88	14.11 ^a^ ± 1.78	19.55 ^b^ ± 2.31	39.67 ^d^ ± 3.05	28.99 ^c^ ± 3.43	29.24 ^c^ ± 3.77	58.67 ^e^ ± 4.15

The specific polyphenols are expressed as micrograms per gram of dry weight (µg/g dw). D.S. represents the standard deviation (*n* = 3). The different letters show significant differences (*p*-value < 0.05).

## Data Availability

Data are contained within the article.
